# Lifestyle preferences drive the structure and diversity of bacterial and archaeal communities in a small riverine reservoir

**DOI:** 10.1038/s41598-020-67774-0

**Published:** 2020-07-09

**Authors:** Carles Borrego, Sergi Sabater, Lorenzo Proia

**Affiliations:** 1 Catalan Institute for Water Research (ICRA), Scientific and Technological Park of the University of Girona, Girona, Spain; 2 Group of Molecular Microbial Ecology, Institute of Aquatic Ecology, University of Girona, Girona, Spain; 3 GRECO, Institute of Aquatic Ecology, University of Girona, Girona, Spain; 4 BETA Technological Center, University of Vic and Central University of Catalonia (UVic-UCC), Vic, Spain

**Keywords:** Ecology, Microbiology, Environmental sciences

## Abstract

Spatial heterogeneity along river networks is interrupted by dams, affecting the transport, processing, and storage of organic matter, as well as the distribution of biota. We here investigated the structure of planktonic (free-living, FL), particle-attached (PA) and sediment-associated (SD) bacterial and archaeal communities within a small reservoir. We combined targeted-amplicon sequencing of bacterial and archaeal 16S rRNA genes in the DNA and RNA community fractions from FL, PA and SD, followed by imputed functional metagenomics, in order to unveil differences in their potential metabolic capabilities within the reservoir (tail, mid, and dam sections) and lifestyles (FL, PA, SD). Both bacterial and archaeal communities were structured according to their life-style preferences rather than to their location in the reservoir. Bacterial communities were richer and more diverse when attached to particles or inhabiting the sediment, while Archaea showed an opposing trend. Differences between PA and FL bacterial communities were consistent at functional level, the PA community showing higher potential capacity to degrade complex carbohydrates, aromatic compounds, and proteinaceous materials. Our results stressed that particle-attached prokaryotes were phylogenetically and metabolically distinct from their free-living counterparts, and that performed as hotspots for organic matter processing within the small reservoir.

## Introduction

Spatial heterogeneity of river networks results from the sequence of lotic segments—which promote the transport and quick transformation of materials—and lentic segments such as large pools and wetlands—which mostly contribute to the process and storage of organic matter^1,2^. This spatial heterogeneity imprints a differential processing associated to their respective water residence times^3^. Inflowing sediments, organic matter, and nutrients from lotic segments are retained and processed in large pools and reservoirs, from where they are subsequently transported downstream^3,4^. These lentic systems may range in size and water retention time, sometimes imposed by human infrastructures. Large reservoirs hold water masses standing for months or years^5^, while small weirs favor shallow waterbodies with shorter water residence times and closer proximity between all potential habitats. In small reservoirs, water and sediments may easily mix in some areas, but sediments accumulate in the deeper parts, collecting both autochthonous and allochthonous organic matter^6^.

Irrespectively of their small size, artificial lentic waterbodies may account for a variety of habitats and resource opportunities for prokaryotes. At the inlet of the small reservoirs, fluvial conditions cause the benthic compartment to prevail as the net receiver of the upstream material. At the lacustrine part, the water column gains progressive relevance with respect to the littoral and deeper sediments. This environmental variability may be translated into identifiable prokaryote lifestyles, including free-living prokaryotes as well as those associated to particles, either in suspension (i.e. organic aggregates) or settled in the streambed (i.e. sediments). These lifestyles account for specific diversity and activity patterns, which have extensively been studied for bacterial communities^7–15^. Most of these studies have revealed that suspended organic aggregates not only harbor more diverse and active bacterial communities than their free-living counterparts, but also sustain a characteristic subset of taxa with special carbon processing capabilities^11,13,14^. Lastly, biofilm bacterial communities in the sediments contribute to the decomposition of organic matter as well as to other relevant biogeochemical processes^16^.

Less information is available for the lifestyle preferences in Archaea. A study conducted in the Arctic shelf showed that archaeal communities attached to particles were different from those free-living in coastal locations, but similar in riverine and ocean sites^17^. A link between organic particles and some marine archaea was revealed by Orsi and co-workers, who unveiled that Marine Group II Euryarchaeota overcame oligotrophic conditions by attaching to suspended organic aggregates^18^. Though these are evidences that some marine archaeal groups may behave similarly to aquatic bacteria regarding lifestyle preferences, we ignore if this is the case in riverine archaeal communities despite the current large body of knowledge^19–21^.

This paper investigates whether the structure and metabolic diversity of bacteria and archaea substantially differed within a small reservoir (tail, mid, and dam sections) and life-styles [free-living (FL), particle-attached (PA), and sediments (SD)]. We assumed that the resulting structure of bacterial and archaeal communities would result from the combination of unlimited dispersal, resulting from the small size and absence of barriers within the system^22^, together with the moderate variation in its local environmental conditions^23^. Both bacterial and archaeal metacommunities could therefore derivate from taxa already present at the systems inlet (i.e. the tail) and transported through the reservoir. In the manner already observed within larger riverine ecosystems, these prokaryotic assemblages would probably take the form of OTUs subsets, with a large part shared between all the habitats^24,25^. Also, and assuming that the longitudinal transport in our small waterbody would be small compared to that occurring in large rivers^25^, we expected a reduced environmental filtering due to the similar conditions characterizing the different habitats. To test this general hypothesis, we characterized the FL, PA, and SD prokaryotic communities within the small reservoir, and related their diversity to their respective potential functional capabilities. We combined targeted-amplicon sequencing of bacterial and archaeal 16S rRNA genes in the bulk (DNA) and potentially active (RNA) community fractions from FL, PA, and SD lifestyles, followed by imputed functional metagenomics to unveil differences in the potential metabolic capabilities. We tested the following specific hypotheses: (i*)* the environmental homogeneity within the reservoir would determine minor compositional differences on the prokaryotic assemblages; (ii) bacterial and archaeal communities would show a different composition according to their lifestyle preferences; and (iii) the functional capabilities of bacterial and archaeal communities would also be related to their lifestyle. Our observations are transferable to systems of similar size being scattered throughout river networks, and under favorable conditions of high temperature and low turbulence.

## Results

### Physical and chemical characteristics of the studied stream

Most physical variables experienced a slight longitudinal change from tail to dam. Conductivity and dissolved oxygen decreased towards the dam while turbidity increased (Table 1). Light reached the stream bottom in the tail but not in the other two locations. Water chemistry was not showing large differences, though DIC slightly decreased towards the dam, and P-PO_4_ remained similar (Table 1). Inorganic N concentration progressively decreased towards the dam and shifted towards higher proportion of less oxidized forms (Table 1). However, most N was in the organic form, and proportion was higher towards the dam (where it accounted for the 82.8%), though TN concentration decreased. The DOC also had an increasing pattern, though concentrations were within a close range. The profiles of the physical and chemical variables in the three sites indicate moderate changes in depth in this small water body (Supplementary Table S1). The same applies to the concentration of chlorophyll *a* along both the longitudinal axis (Table 1) and in depth the three sites (Suppl. Table S1).

**Table 1:** Average (± standard deviation) values for the main physical and chemical variables as well as Chl *a* concentration at the three sites of the studied stream.

Variable	Site		
	Tail	Mid	Dam
Max. depth (m)	0.60	3.90	2.70
Temperature (ºC)	16.9 ± 0.1	16.1 ± 0.05	16.5 ± 0.24
Conductivity (µS cm^–1^)	522 ± 1.2	505 ± 0.05	462 ± 11.4
Oxygen (mg/L)	9.4 ± 0.1	7.7 ± 0.05	6.8 ± 0.2
Oxygen (% sat)	96.9 ± 1.4	78.3 ± 0.05	70.1 ± 1.8
pH	8.2 ± 0.01	8.2 ± 0	8.1 ± 0.01
PAR (µE cm^–2^ s^–1^)	58.0 ± 49.4	134 ± 283	18.9 ± 24.8
Turbidity	6.3 ± 0.5	16.6 ± 4.7	16.6 ± 2.31
Na (mg L^–1^)	8.3 ± 0.05	7.9 ± 0.03	6.7 ± 0.03
K (mg L^–1^)	1.7 ± 0.01	1.7 ± 0.02	1.9 ± 0.07
Mg (mg L^–1^)	14.3 ± 0.89	13.6 ± 0.13	23.9 ± 10.1
Ca (mg L^–1^)	77.7 ± 2.9	80.2 ± 4.8	76.8 ± 4.8
Cl (mg L^–1^)	13.3 ± 0.06	12.8 ± 0.01	10.2 ± 0.01
S-SO_4_ (mg L^–1^)	10.3 ± 0.01	10.1 ± 0.01	8.5 ± 0.005
F (mg L^–1^)	0.07 ± 0.004	0.06 ± 0.0	0.06 ± 0.001
DIC (mg L^–1^)	62.7 ± 1.17	63.3 ± 0.17	59.4 ± 0.44
DOC (mg L^–1^)	1.38 ± 0.03	1.47 ± 0.04	1.88 ± 0.01
N-NH_4_ (mg L^–1^)	0.01 ± 0.05	0.02 ± 0.003	0.04 ± 0.005
N-NO_2_ (mg L^–1^)	0.02 ± 0	0.02 ± 0	0.03 ± 0
N-NO_3_ (mg L^–1^)	4.36 ± 0.001	4.14 ± 0.003	3.16 ± 0
TN (mg L^–1^)	20.9 ± 3.8	20.1 ± 2.2	18.8 ± 0.5
P-PO_4_ (mg L^–1^)	0.02 ± 0	0.02 ± 0.001	0.02 ± 0.001
TP (mg L^–1^)	0.04 ± 0.01	0.05 ± 0.002	0.10 ± 0.03
Chl *a* (µg L^–1^)	2.32 ± 0.14	2.49 ± 0.23	2.59 ± 0.09

### Composition of bacterial communities

**Figure 1:**
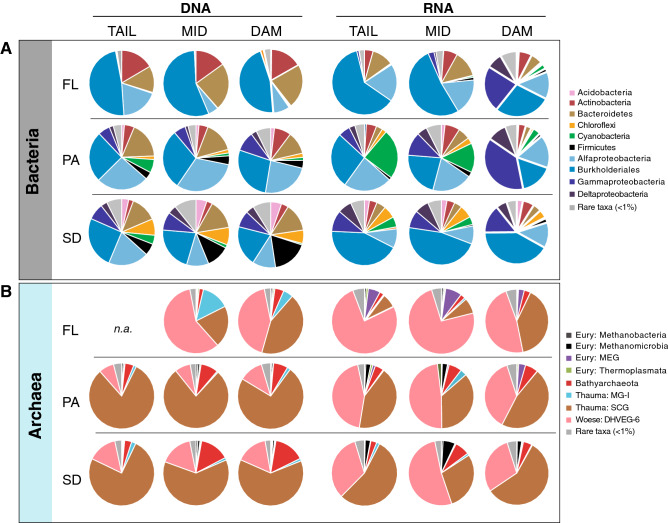
Relative abundance (in % of total reads) of (**A**) bacterial phyla (classes for the Proteobacteria and order for the Burkholderiales, formerly known as Betaproteobacteria and now within the Gammaproteobacteria), and (**B**) archaeal phyla (classes for the Euryarchaeota, Thaumarchaeota and Woesearchaeota) across stream sites (tail, mid, down; as columns) and lifestyles [free-living (FL), particle-attached (PA) and sediment (SD), as rows] in DNA (left) and RNA (right) libraries.

**Figure 2:**
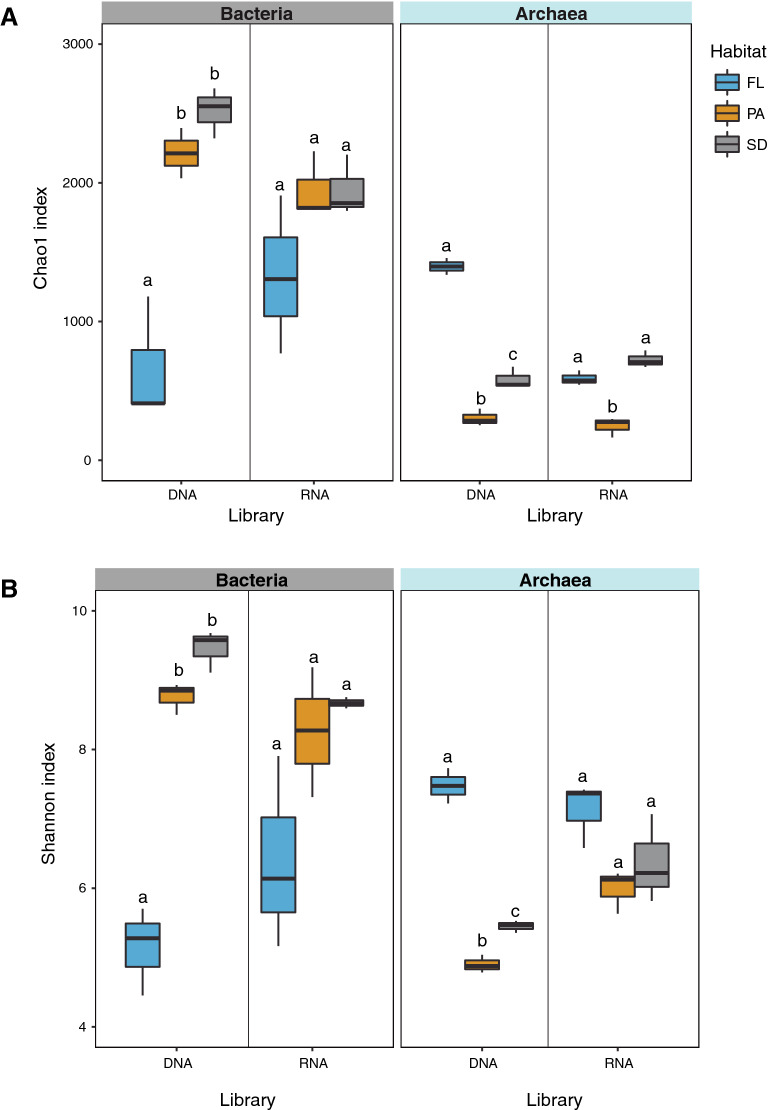
Comparison of (**A**) Chao1 and (**B**) Shannon diversity estimators for bacterial and archaeal communities in DNA and RNA libraries according to their lifestyle preferences. The lower and upper edges of each boxplot are the first and third quartiles, the midline shows the median and the whiskers extend from the minimal to the maximal values. Different letters above boxplots indicate significant differences (α = 0.05).

Bacterial communities in the bulk fraction of FL, PA, and SD were dominated by sequences affiliated to the class Alpha proteobacteria and the order Burkholderiales (within the class Gammaproteobacteria). This was consistent within the reservoir (upper panel in Fig. 1, left side). Sequences affiliated to phyla Actinobacteria and Bacteroidetes were also prevalent in the FL community, but their relative contribution (especially in the case of the Actinobacteria) was small in the SD and PA samples. Bacterial communities in the sediment were particularly enriched in sequences affiliated to the Firmicutes, which increased in relative abundance towards the dam. The composition of bacterial communities in RNA libraries (assumed as being the active fraction of the communities) differed with respect to those from the DNA libraries (and included Actinobacteria and Bacteroidetes in FL samples, Cyanobacteria and the Gammaproteobacteria in PA samples, and Burkholderiales and the Firmicutes in the sediment) (Fig. 1, upper panels). Overall, free-living bacterial communities in the DNA fraction (though not in the RNA fraction) were significantly less rich and diverse than those attached to particles or inhabiting the sediment (Fig. 2). The differences in alpha diversity estimators between nucleic acid fractions and communities across the longitudinal axis disappeared (Supplementary Fig. S1). This was an indication that lifestyles and not collection sites were the key factor structuring bacterial communities of this waterbody (see below).

The less pronounced differences in Chao1 and Shannon indices in the RNA fraction (Fig. 2) suggested that (i) prevalent phyla in the bulk bacterial community were less represented in its active fraction (e.g. Actinobacteria in FL or Bacteroidetes and Firmicutes in PA and SD habitats, Fig. 1A), and (ii) rare groups in the bulk community (i.e. Gammaproteobacteria in all habitats, Deltaproteobacteria in PA and SD) turned up to be relevant in the active fraction. The changes in the rank of abundance of the ten most common bacterial genera in FL, PA, and SD bacterial communities further supports the observed divergence between bulk and active fractions (Suppl. Fig. S2A). Sequences affiliated to genus *Flavobacterium*, *Pseudarcicella,* and *Candidatus* Aquiluna, decreased their contribution in the active fraction though were largely represented in the bulk fraction of FL samples. However, members of the genus *Limnohabitans* showed similar contributions in the bulk and active fractions of the FL community. The active fraction of PA bacterial communities had a high relative abundance of unclassified sequences within the Proteobacteria and Cyanobacteria, and sequences affiliated to the OM27 clade within the class Deltaproteobacteria (Suppl. Figure S2A). This observation suggested that the particle-attached bacterial communities maintained a large reservoir of active but uncultured unknowns.

**Figure 3:**
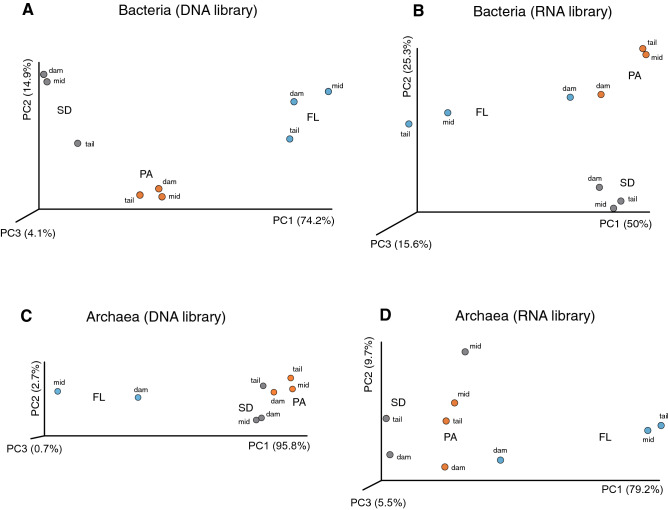
PCoA ordination of bacterial (**A**, **B**) and archaeal (**C**, **D**) communities based on weighted UniFrac distance of phylogenetic relatedness. Samples are color coded according to the habitat they belong to (blue: Free-living; orange: particle-attached; gray: sediment).

The beta-diversity analysis of the bacterial communities confirmed that differences along the spatial axis (i.e. tail, mid, dam) were non-significant (PERMANOVA, pseudoF = 0.174, *p* = 0.904), but significantly changed between lifestyles (FL-PA-SD; PERMANOVA, pseudoF = 18.26, *p* = 0.006). Analogous results were obtained for the RNA library (PERMANOVA, *p* = 0.507 and *p* = 0.004 for sites and lifestyles, respectively). Ordination of samples according to their phylogenetic relatedness (i.e. weighted UniFrac distance) clustered the bacterial communities by their lifestyle preferences but not by the collection sites (Fig. 3A). Further, communities from PA and SD habitats were more phylogenetically related to each other than to their FL counterparts; again, similar results were obtained for the RNA fraction (Fig. 3B). In this latter case, FL and PA from the dam grouped closely indicating that shared a common set of active groups (i.e. Gamma- and Deltaproteobacteria).

**Figure 4:**
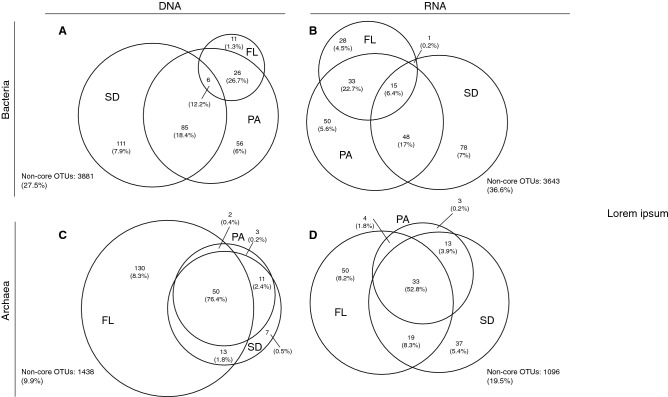
Distribution of specific and shared OTU between lifestyles (FL: Free-living, PA: Particle-attached, SD: Sediment) in bacterial (**A**, **B**) and archaeal (**C**, **D**) communities from DNA (**A**, **C**) and RNA (**B**, **D**) libraries. Numbers in brackets correspond to the average relative abundance of the identified OTUs in that habitat. The area of each circle is proportional to the number of OTUs in each compartment.

PA and SD shared 85 of the core OTUs (representing the 18% of total OTUs in the bulk community), while PA and FL only shared 26 OTUs (26.7% of the total) (Fig. 4A). Analogous patterns were obtained for the RNA fraction (Fig. 4B). Those OTUs contributing less than 0.05% (non-core OTUs) outnumbered core OTUs in both libraries (3881 and 3643 in DNA and RNA fractions, respectively). This result evidences the large contribution of rare taxa (27.5% and 36.6% of non-core OTUs in DNA and RNA fractions, respectively). Only a few OTUs were shared between all three habitats (cosmopolitan OTUs): 6 OTUs, 12.2% in the DNA, 15 OTUs, 6.4% in the RNA.

### Composition of archaeal communities

Archaeal communities were dominated by sequences affiliated to Deep Hydrothermal Vent Euryarchaeota Group 6 (DHVEG-6, Woesearchaeota) and Soil Crenarchaeota Group (SCG, Thaumarchaeota) (Fig. 1, bottom panels). The SCG is composed of terrestrial ammonia-oxidizing archaea (AOA) and accounted for the 77% in the PA and 67% in the SD. In the RNA fraction, the relative abundance of sequences affiliated to the DHVEG-6 largely increased, while those abundant in the DNA largely decreased (Fig. 1, bottom right panel). The sequences affiliated to the phylum Bathyarchaeota also decreased in the RNA fractions, while the Miscellaneous Euryarchaeota Group (MEG) showed a reversed pattern. The sequences affiliated to methanogenic archaea were below 1% of total archaeal reads of the SD, and only reached 2% in the RNA fraction. The differences between bulk and potentially active fractions of the archaeal communities was consistent amongst the top ten abundant classes (Suppl. Figure S2B).

Free-living archaeal communities were richer and more diverse than those attached to particles or inhabiting the sediment (Fig. 2, right panels). This difference was not so substantial in the RNA fraction, suggesting that bulk archaeal communities contained a large proportion of non-active members from a few taxa (i.e. SCG). Non-significant differences of alpha diversity existed between the sites (Suppl. Fig. S1, right panels). Differences in the beta-diversity of the DNA and RNA fractions were only significant between lifestyles (PERMANOVA on lifestyle, *p* = 0.002 and *p* = 0.01, respectively; PERMANOVA on site, *p* = 0.765 and *p* = 0.594, respectively). Ordination of the samples using the weighted UniFrac distance showed a close phylogenetical relationship between the archaeal communities of PA and SD (Fig. 3C, D).

The archaeal core OTUs were more numerous in the FL than in the PA and SD, both for the DNA and RNA fractions (Fig. 4C, D). The archaeal communities were characterized by a high number of cosmopolitan OTUs, common to the three habitats (50 and 33 in DNA and RNA fractions, respectively). In clear contrast with the patterns within the bacterial communities, the cosmopolitan archaeal OTUs were dominant (76.4% and 52.8% in DNA and RNA fractions, respectively) than those specific for a given lifestyle or the non-core OTUs (Fig. 4C, D). OTUs affiliated to Woesearchaeota (DHVEG-6) were prevalent in all lifestyles except in the DNA fraction of PA and SD which were mainly dominated by the Thaumarchaeota (SCG) (Fig. 1).

### Lifestyle preference and indicator OTUs

**Figure 5:**
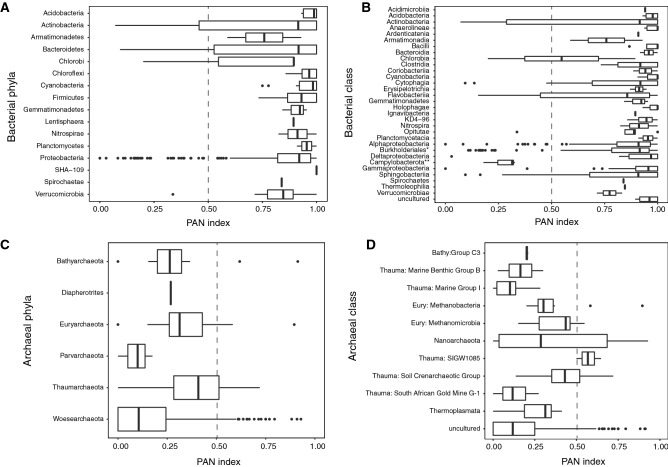
Boxplots showing the particle-associated index (PAN index) for OTUs grouped according to (**A**) bacterial phyla, (**B**) bacterial classes (order for Burkholderiales), (**C**) archaeal phyla, and (**D**) archaeal classes. The lower and upper edges of each boxplot are the first and third quartiles, the midline shows the median and the whiskers extend from the minimal to the maximal values. The vertical dotted line shows a PAN index of 0.5. *formerly known as Betaproteobacteria, now an order within the Gammaproteobacteria^61^; **formerly known as Epsilonproteobacteria, now reclassified as new Phylum^61^.

The PAN indexes at Phyla or Class levels indicated that most bacterial OTUs preferred the PA lifestyle (average PAN = 0.829 ± 0.249) (Fig. 5A, B), while the archaeal OTUs had an opposite trend (average PAN = 0.210 ± 0.21) (Fig. 5C, D). OTUs associated to the FL lifestyle were affiliated to genus *Limnohabitans*, *Polynucleobacter,* and *Variovorax* (order Burkholderiales, Suppl. Fig. S3). For Archaea, the only OTUs with PAN index values across the 0.5 line were affiliated to the class Nanoarchaeota (phylum Woesearchaeota), which had an equal preference for PA and FL lifestyles (Fig. 5D).

The IndVal index of bacterial indicator OTUs identified up to 327 OTUs in the bulk DNA fraction and 280 in the RNA fraction for all lifestyles (FL, PA, SD) and community fractions (DNA and RNA). The SD habitat had the largest number of indicator OTUs (76.8% and 69.6% in DNA and RNA libraries, respectively), mostly affiliated to phyla Bacteroidetes, Chloroflexi, Firmicutes, Acidobacteria, and classes Gammaproteobacteria (including the Burkholderiales) and Deltaproteobacteria (Suppl. Fig. S4A). OTUs of these proteobacterial classes were also the most abundant in the RNA fraction (Suppl. Fig. S4B). Indicator OTUs for the PA communities affiliated to classes Alpha- and Gammaproteobacteria and to phyla Bacteroidetes and Actinobacteria (Suppl. Fig. S4A, B). The indicator OTUs for the FL lifestyle mainly affiliated to genus *Limnohabitans*, *Polynucleobacter,* and *Variovorax* (order Burkholderiales within the Gammaproteobacteria, Suppl. Fig. S4A, B), which were also identified as typical FL taxa through the PAN index (Suppl. Fig. S3).

The IndVal analysis of bulk archaeal communities (DNA fraction) obtained indicator OTUs only for the FL lifestyle, affiliated to the Woesearchaeota (DHVEG-6) (Suppl. Fig. S4C). This agreed with the dominance of OTUs under PAN index values below 0.5. Indicator OTUs for the PA and the SD lifestyles were only identified in the RNA fraction of archaeal communities and were mainly affiliated to the DHVEG-6, the Methanomicrobia, and the Thaumarchaeota (Suppl. Fig. S4D).

### Functional profile of bacterial communities

We were limited by the fraction that could be mapped to KEGG organisms (FTU) when aiming to assess the potential capacity to degrade dissolved organic matter. This fraction ranged from 40 to 60% OTUs in the bacteria and from 2 to 15% in the archaeal OTUs (data not shown). In particular, the archaeal communities were composed by a large fraction of uncultured representatives, and this limited the validity of any further functional prediction. Assuming this limitation, we restrained our prediction to the functional capabilities of bacterial communities.

**Figure 6:**
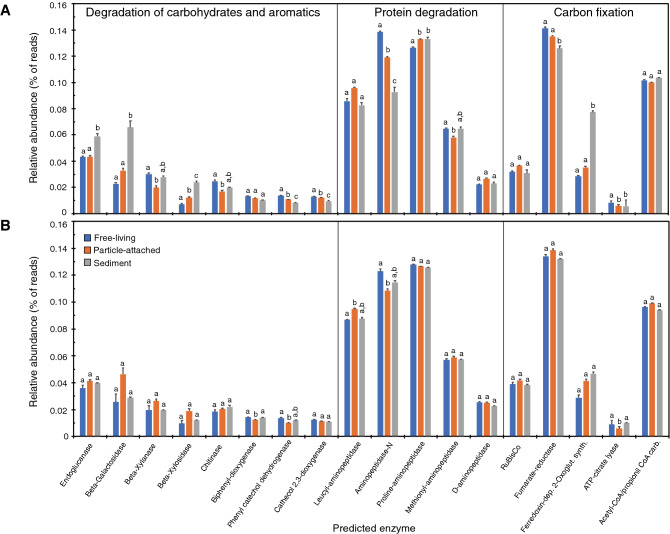
Changes in the relative abundance of predicted genes [expressed as the average in each habitat (± SD)] from (**A**) the DNA fraction and (**B**) the RNA fraction of bacterial communities grouped according their lifestyle preferences. Results from post-hoc Tukey HSD test (or its non-parametric equivalent when appropriate) for each gene are shown in letters above bars after adjustment of *p*-values for multiple comparisons (see Material and Methods for details). Genes are grouped by the metabolic pathway their belong to.

Predicted functional profiles of bacterial genes involved in the degradation of carbohydrates, aromatic compounds, and proteins, as well as in the carbon fixation, are shown in Fig. 6. The bulk bacterial communities in sediments showed a higher number of genes involved in the degradation of complex carbohydrates and aromatics (e.g. endoglucanase, beta-galactosidase, and beta-xylosidase) (Fig. 6A). The genes involved in the degradation of proteinaceous material were evenly distributed across compartments, but aminopeptidase-N was significantly higher in FL. The functional profiles predicted after the RNA fraction were similar in the three habitats (Fig. 6B).

## Discussion

Longitudinal changes in composition of microbial communities have been observed in large river systems^24^ or related to river continuity interruption by dams^12,25^. The size of the lentic waterbody and its water residence time may certainly contribute to potential differences. The lentic waterbody which was analyzed show a high physical and chemical homogeneity along the longitudinal (Proia et al.^3^, Table 1) and vertical axes, as well as a short (1.5–2 days) water residence time. These conditions favored the similarity from tail to dam of the prokaryotic assemblages, both regarding their composition and functional metabolic profiles. In clear contrast, we found striking disparities in the structure of bacterial and archaeal communities in relation to their lifestyle preferences. The structure of these assemblages agreed with a large repertoire of phylogenetic and metabolic adaptations of aquatic prokaryotes to organic matter sources, either dissolved or particulate^9,26^.

In aquatic systems, particulate organic matter forms micro- and macroaggregates (the so-called marine, river or lake snow^27^).These act as nutritional hot spots for phylogenetically diverse and metabolically versatile bacterial communities^8,11,15,28–32^. Regarding archaeal communities, similar responses have been described in the coastal Pacific ocean^18^ and the Arctic shelf ecosystem^17^. All these are compelling evidences that particle-attached microbes are phylogenetically and metabolically distinct from their free-living counterparts. This was the case also in our system, where both bacterial and archaeal communities attached to particles segregated from those free-living and remained closer—in phylogeny and structure (shared OTUs)—to the benthic communities (SD). However, functional differences between were less apparent and indicated that FL and PA communities shared a common set of active groups with similar nutritional cues. Remarkably, the functional profile of the bacterial communities in the sediment was not different from those in the planktonic compartment (PA and FL lifestyles). It was likely that the functional hallmarks of bacteria attached to sediment particles could eventually blur because of sediment resuspension and particle settlement (two processes probably very active in such a small system).

Some studies have outlined that particle-attached bacterial communities were less rich and diverse than free-living ones^33,34^, while the opposite trend has also been reported^11,31,32,35,36^. One of the most relevant observations in this study was that bacterial and archaeal communities had opposite trends in richness and diversity. This was a common pattern to the free-living and particle-attached assemblages, in both DNA and RNA fractions (Fig. 2). Such a contrasting pattern has been also observed in marine bacterial and archaeal communities, related to the size of suspended particles in the water column^37^. It may be argued that preferences towards particles of different size may trigger distinct colonization strategies and subsequent specific composition of bacteria or archaea, also related to their metabolic capacities^37^. In this regard, the complex genetic repertoire required to the PA lifestyle^38,39^ seems to be evolutionary maintained in certain groups of prokaryotes, and this may favor their adaptation to such lifestyle^13^. Similar adaptations are expected for taxa preferentially associated to the FL lifestyle, with rare transitions between PA and FL habitats^13^. Accordingly, the different behavior of bacterial and archaeal communities in terms of richness and diversity probably respond to their phylogenetic structure rather than to environmental cues. Whereas bacterial communities in PA (and SD) are very diverse and composed by groups usually enriched in suspended particles^11,15,31,32,37^, the low diversity of archaeal communities in PA (and SD) respond to the overwhelming dominance of SCG Thaumarchaeota. The enrichment of free-living archaeal communities in members of the Marine Group-I Thaumarchaeota (an autotrophic AOA usually associated to the FL lifestyle^13^) and the Woesearchaeota (see below) accounts for the higher diversity observed in FL compared to PA and SD communities.

Overall, our results support that certain bacterial and archaeal groups adhere either to FL or PA lifestyles^11,15,31,32,37^. Still, some particularities deserve to be highlighted. Members of the genus *Limnohabitans* (Order Burkholderiales) preferred a free-living lifestyle, and this agrees with their dominance in freshwater ecosystems worldwide^40–43^. The high abundance of Bacteroidetes in the bulk FL communities contrasted to their well-known preference to grow attached to suspended organic particles^11,14,31,32^. Finally, the most abundant group of sequences in the RNA fraction of the PA bacterial communities were affiliated to uncultured Proteobacteria, indicating an active but unknown pool of metabolic capabilities. This large number of non-core OTUs probably corresponds to taxa “passing through” the system^44^, a likely influence of the neighbouring terrestrial habitats influence^16^.

The archaeal communities in our small waterbody were dominated by sequences affiliated to the Soil Crenarchaeota Group (Thaumarchaeota) and the DHVEG-6 (Woesearchaeota^45^). The Soil Crenarchaeota Group encompasses ammonia oxidizing archaea of terrestrial and sedimentary environments, where they are main contributors to nitrification^46^. The SCG archaea presumably may carry out ammonia oxidation linked to autotrophic or mixotrophic growth, both in the sediment and in the suspended organic particles. Members of the Woesearchaeota are widespread in freshwater and marine habitats (including sediments), groundwater, soil, wastewater, and extreme environments^47^. The Woesearchaeota exhibit a large intragroup phylogenetic diversity (up to 26 subgroups proposed so far^47^), that points out to some sort of habitat segregation. A meta-analysis on diverse sequence datasets has revealed that the oxic status of the source environment is a major factor shaping their community composition^47^. This group was dominant (83% of total OTUs) in the water column of fully oxic, oligotrophic Pyrinean lakes^48^. The oxic conditions prevailing in our lentic waterbody from the tail to the dam probably favoured their dominance within the archaeal communities. Besides, the overwhelming occurrence of woesearchaeotal sequences in the RNA fraction in all habitats, suggests their active contribution to organic carbon cycling. Current knowledge on their metabolic capacities of suggests that Woesearchaeota have an anaerobic heterotrophic lifestyle with several metabolic deficiencies, to be complemented with potential syntrophic interactions with bacterial or archaeal (e.g. methanogens) counterparts^47^. Finally, a surprisingly low relative abundance of Bathyarchaeota and Thermoplasmata members in the sediment contradicts their assumed condition of core-generalists for this habitat^49^. Although they are prevalent in organic rich marine and freshwater sediments worldwide^50^, the lack of euxinic conditions (anoxia and sulfide) in our lentic waterbody might explain their low representativeness in comparison to lacustrine sediments^51,52^.

## Material and methods

### Study site

A small lentic system in the Fluvià River was selected to investigate the structure and functional dynamics of the microbial communities. The Fluvià is a permanent watercourse spotted by small dams from headwaters to the mouth. It flows 97 km from the Eastern Pyrenees to the Mediterranean Sea (northeastern Iberian Peninsula), and has a catchment area of 990 km^2^, mostly covered by mixed forests (78%), agricultural (19%) and urban (3%) lands. The climate is typically Mediterranean, with warm temperatures and moderate rainfall. The lentic segment was located upstream to the city of Olot (30,000 inhabitants) and is the first occurring along the Fluvià river course (decimal degree coordinates 42.174738, 2.470470). The studied lentic segment was 900 m long, 2.4 m of mean depth, and had a maximum depth of 5.4 m. Its volume was 17,434 m^3^ and its surface area 14,212 m^2^. The sediments covering the streambed were mostly silt and clays. We defined three different sites along the lentic waterbody, which included an upstream section or tail, a medium lentic part, and the proximity of the dam (Suppl. Figure S5).

### Sampling and physical and chemical analyses

The sampling was performed at the end of summer 2013, when the low water flow coincided with the most productive period^3^. Physical and chemical water characteristics in the water column and sediments were measured simultaneously.

#### Sample collection

All in-situ measurements and collection of samples were carried out from a pneumatic boat. We used portable probes to measure the temperature and conductivity (Cond 3310; WTW, Weilheim, Germany), pH (pH 3110; WTW) and dissolved O_2_ concentration (YSI ProODO Handheld; Yellow Springs Instruments, Yellow Springs, Ohio) of surface water. Water samples for dissolved organic carbon (DOC) and dissolved inorganic carbon (DIC), anions, and cations were collected in triplicate. Samples were filtered through pre-combusted (4 h at 450 °C) GF/F and pre-rinsed 0.7 µm glass-fiber filters (Whatman, Maidstone, UK), and stored in 125 mL bottles. Vertical profiles of the main physical and chemical variables (and Chlorophyll-a) were also performed in the three sites (tail, mid, dam).

Water column samples for biological examination were collected in the 0.5–1 m depth in the three sites. We collected triplicate samples of water in each of the sites using 2.5 L sterile plastic bottles. The PA fraction was separated from the FL planktonic fraction by filtering water samples through 5 µm and 0.2 μm pore-diameter nylon membrane filters^53^. Sediments were collected with a bottom grab sampler (Ekman–Birge type; Hydro-Bios, Kiel, Germany) by triplicate, from which we collected 3.14 cm^3^ of superficial sediment (1 cm depth) using an untapped 25 mL plastic syringe (2 cm diameter). Sediments were later distributed into vials for specific measurements of microorganisms inhabiting the surficial sediment and stored at 4 ºC in 30 mL glass vials with 4 mL of 0.2 μm-filtered river water (nylon membrane filters). All samples were stored at 0 ºC in a portable icebox and later frozen at − 20 ºC at the arrival to the laboratory (2 h after collection).

#### Physical and chemical measurements

DIC and DOC were measured with a Total Organic Carbon Analyzer (TOC-VCSH; Shimadzu, Kyoto, Japan). We measured anions and cations with ion chromatography (model IC5000; DIONEX, Sunnyvale, California) following UNE-ENISO 10304–1 (1995) and UNE-EN ISO 14911 (2000), respectively. We collected 3 replicates of unfiltered water and stored them for analyses of total N (TN) and P (TP). We measured TN and TP after alkaline digestion of the unfiltered water samples^54^ and subsequent spectrophotometric determination.

### Molecular analyses

#### Nucleic acid extraction

Extraction of RNA and DNA from all samples was carried out using the PowerSoil Total RNA Isolation Kit and RNA Powersoil DNA Elution Accessory Kit, respectively (QIAGEN, MD, USA) following manufacturer instructions. RNA extracts were digested with Turbo DNA-free (Ambion Inc., Austin, TX, USA) to remove traces of residual DNA and retrotranscribed to cDNA using random hexamer primers and SuperScript III First-Strand Synthesis System for RT-PCR (Invitrogen, Carlsbad, CA, USA) according to manufacturer instructions. Nucleic acid concentrations were determined using QUBIT 2.0 Fluorometer (Invitrogen Molecular proves Inc., Oslo, Norway).

#### High-throughput sequencing and processing

DNA and cDNA extracts were subjected to high-throughput multiplexed 16S rRNA gene sequencing with the Illumina MiSeq System (2 × 250 PE) at the facilities of RTLGenomics (Lubbock, TX, USA). Briefly, extracts were used as a template in PCR reactions using primers 28F/519R (Bacteria, regions V1–V3) and Arch519wF/Arch1017R (Archaea, regions V4–V6) complemented with Illumina-adapters and sample-specific barcodes according to RTLGenomics protocols. Raw sequence datasets were pre-processed at RTLGenomics facilities to reduce noise and sequencing artefacts as previously described^55^. Demultiplexing according to sample barcodes, sequence quality assessments, delineation of OTUs (97% cutoff), chimera detection and downstream phylogenetic analyses were conducted in QIIME v1.9.1^56^. In QIIME, representative sequences from each OTU were aligned to the Greengenes imputed core reference alignment^57,58^ using PyNAST^59^. Taxonomical assignments for each OTU were done using the BLAST method and the QIIME-formatted version of the SILVA 132 reference database (10-April-2018)^60^, which incorporates the new arrangement for the Proteobacteria (Burkholderiales (formerly known as class Betaproteobacteria) as an order within the Gammaproteobacteria, and the class Epsilonproteobacteria as a the new phylum Campylobacterota)^61^. For community analysis, the number of sequences in each sample was normalized by randomly selecting a subset of 11,000 sequences (for both bacteria and archaeal datasets) from each sample to standardize sequencing effort across samples and to minimize bias due to different number of total sequences. QIIME was also used to calculate alpha diversity estimators (Chao1 and Shannon) and to compare microbial communities across sites (tail, mid, dam) and life-styles (FL, PA, SD) using the weighted UniFrac distance^62^. In the latter case, differences were assessed for statistical significance using the PERMANOVA test (999 permutations) implemented in QIIME. Rarefied OTU tables generated in QIIME were then uploaded into ampvis2^63^ in R^64^ for the identification and visualization of the ten most abundant OTUs and core OTUs for each lifestyle. The latter were identified in ampvis2 using an abundance threshold of 0.05% (minimal abundance per sample) and a frequency threshold of 80% (presence in 80% of the samples). Ampvis2 was also used to construct Venn diagrams to graphically visualize the core OTUs shared between lifestyles. Raw sequence datasets have been deposited in the NCBI Sequence Read Archive (SRA) database under accession nº (Bioproject PRJNA545687).

#### Calculation of particle-association niche index

To estimate the habitat segregation of a given OTU, we calculated the “particle-association niche index” (PAN index) according to Salazar and co-workers^13^. The PAN index allows positioning each OTU in a continuum describing its lifestyle preference, from completely planktonic (PAN index = 0) to a permanent association to particles (PAN index = 1). Accordingly, we used the abundance-weighted mean for each OTU in a sample while ascribing a value of 0 and 1 to FL and PA samples, respectively (see^13^ for details). Sediment samples were not considered for calculations since they accumulate OTUs settling from the water column (either FL or PA). Calculation of PAN index was finally done on 503 bacterial and 402 archaeal OTUs after discarding all OTU ≤ 10 reads across samples to avoid biases caused by rare OTUs^13^.

#### Identification of indicator OTUs

To identify indicator OTUs for each habitat (FL, PA, SD) we calculated the IndVal index^65,66^. The IndVal index is a combined measure of ‘specificity’ (A, the proportion of the total reads of an OTU that appear in a given size fraction) and ‘fidelity’ (B, the proportion of samples of a given size fraction where an OTU occurs)^66^. The significance of the association was tested using permutation tests. We only retain as “indicator species” those OTUs with IndVal values ≥ 0.8 and *p *values < 0.05.

#### Imputed functional metagenomics

Functional profiles were predicted from 16S rRNA gene sequence datasets using Tax4Fun^67^. Genes involved in the metabolic pathways of interest were identified using their KEGG orthologs (Kyoto Encyclopedia of Genes and Genomes, https://www.genome.jp/kegg/ko.html). Statistical significance of differences in the relative abundance of genes involved in pathways of interest between habitats were assessed using One-way ANOVA (or Kruskal–Wallis Chi-squared test when normality was not achieved). Post-Hoc tests for comparison between levels of factor habitat (FL, PA, SD) were done using Tukey HSD (or Wilcoxon Rank Sum) after adjusting the resulting *p*-values for multiple testing using the False Discovery Rate correction (FDR) with a significance cut-off of 0.05^68^. All tests were conducted in R^64^ using packages *stats*, *car*, and *npar*. Package *ggplot2* was used for data visualization^69^.

## Supplementary information

Supplementary file1 (PDF 8522 kb)
