# Supplementary material for: Lifestyle preferences drive the structure and diversity of bacterial and archaeal communities in a small riverine reservoir
Source: Sci Rep. 2020 Jul 9;10:11288. doi: 10.1038/s41598-020-67774-0 (PMC7347578; doi:10.1038/s41598-020-67774-0)
Supplement: Supplementary file 1 — Supplementary file1 (PDF 8522 kb) [file 41598_2020_67774_MOESM1_ESM.pdf]

# Habitat segregation affects the structure and diversity of bacterial and archaeal communities in a small riverine reservoir

Carles Borrego, Sergi Sabater & Lorenzo Proia

## Supplementary Information

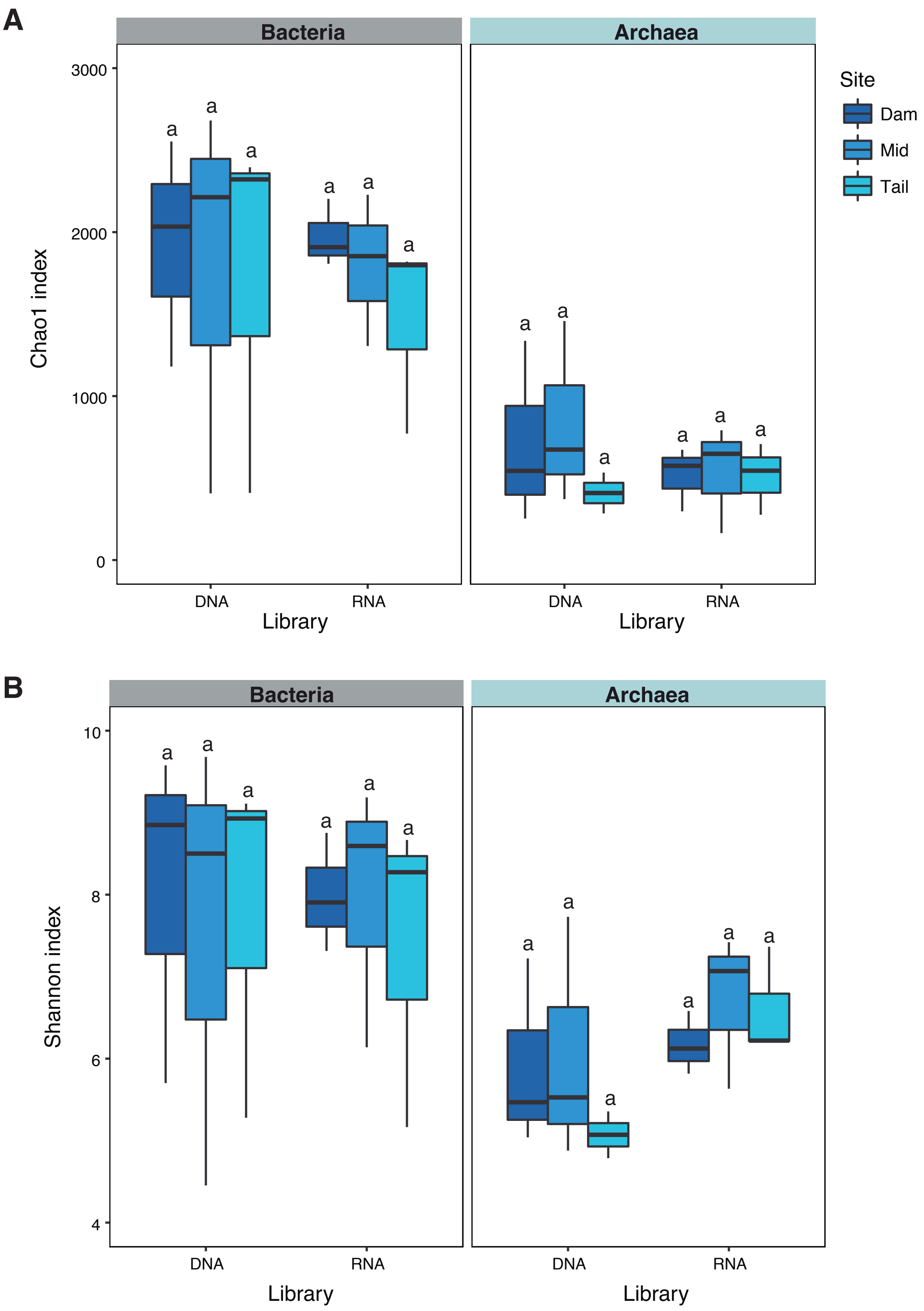


**Supplementary Figure S1.** Comparison of (A) Chao1 and (B) Shannon diversity estimators for bacterial and archaeal communities in DNA (bulk) and RNA (active) libraries according to the site samples were collected from. The lower and upper edges of each boxplot are the first and third quartiles, the midline shows the median and the whiskers extend from the minimal to the maximal values. Different letters above boxplots indicate significant differences ($\alpha = 0.05$).

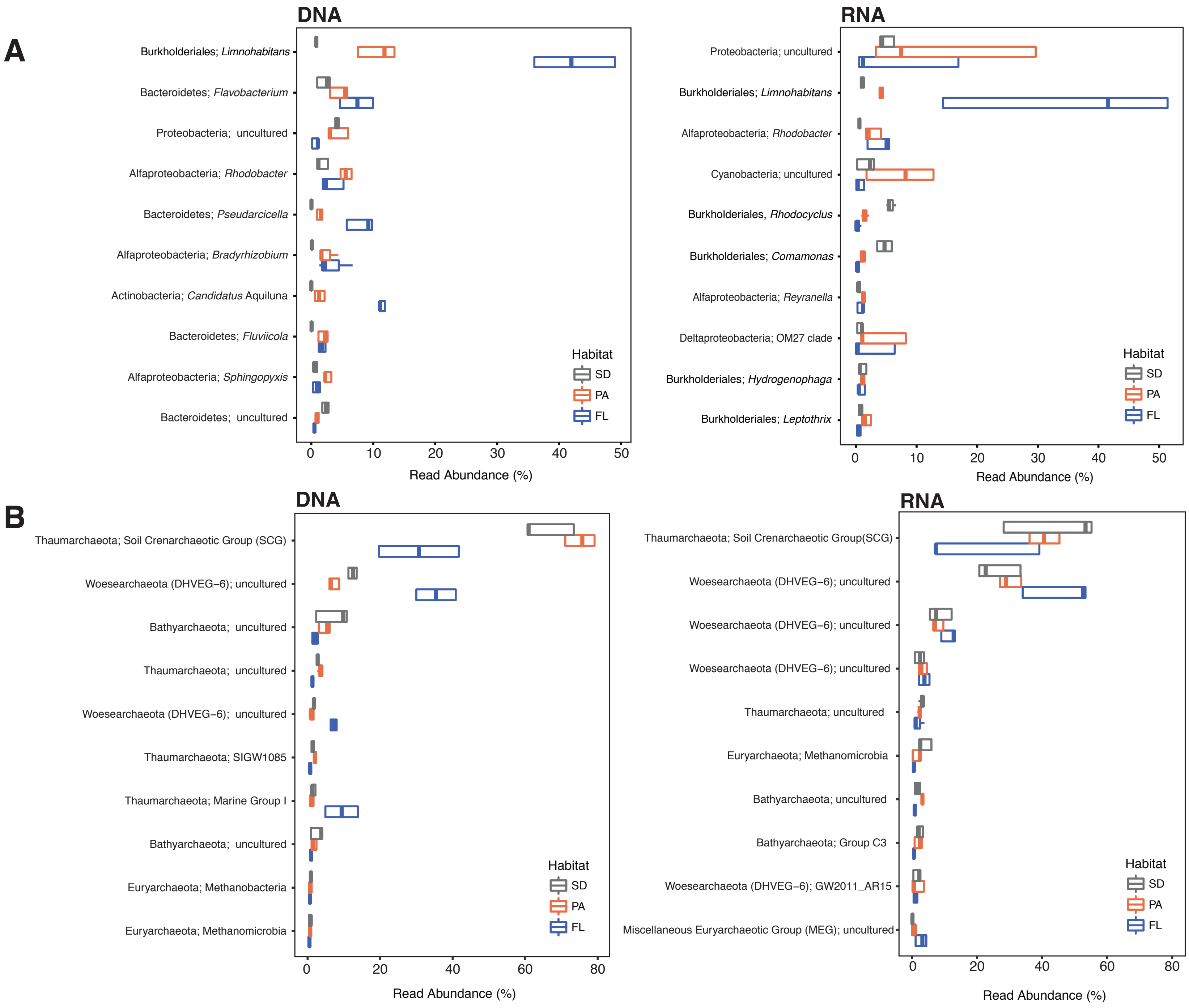


**Supplementary Figure S2.** Relative abundance (in % of total reads) of the top ten bacterial genera (A) and archaeal classes (B) in microbial communities from sediments (SD), aggregates (particle-attached, PA) and the plankton (free-living, FL) in DNA (left) and RNA (right) libraries.

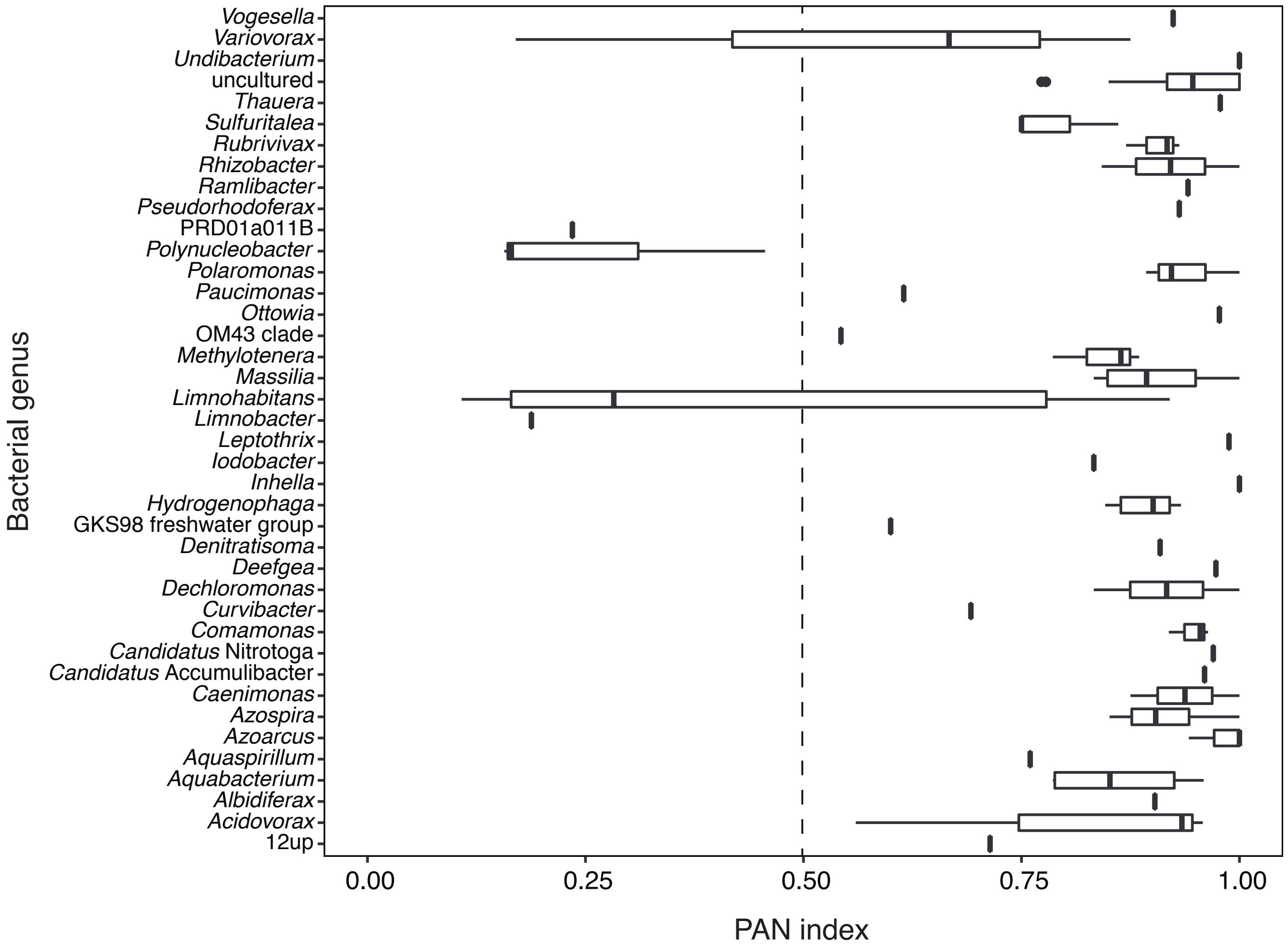


**Supplementary Figure S3.** Boxplots showing the particle-associated index (PAN index) for OTUs affiliated to genera within the order Burkholderiales (class Gammaproteobacteria). The lower and upper edges of each boxplot are the first and third quartiles, the midline shows the median and the whiskers extend from the minimal to the maximal values. The verical dotted line shows a PAN index of 0.5.

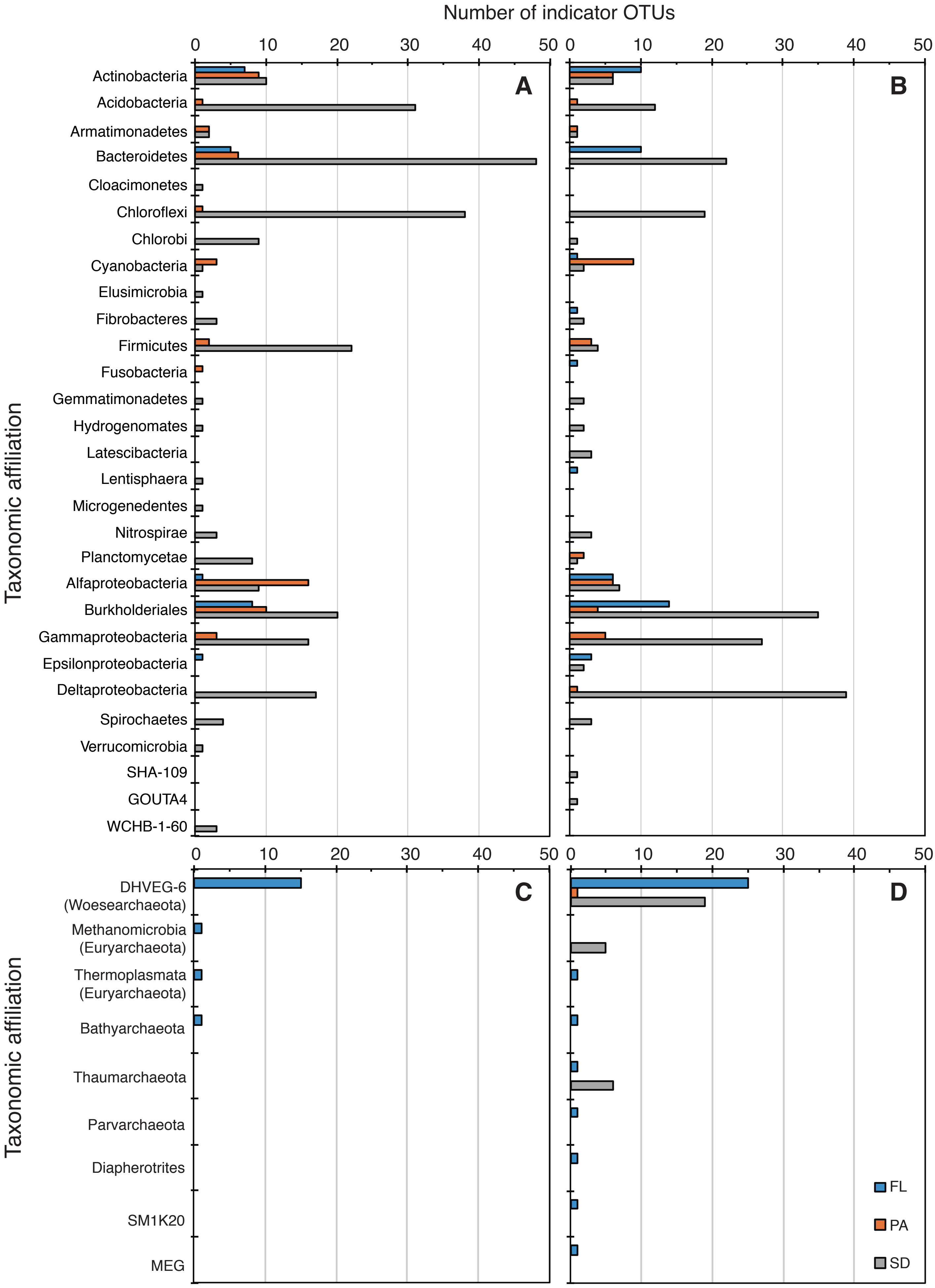


**Supplementary Figure S4.** Taxonomic distribution of the indicator OTUs (indval >0.8 and $p < 0.05$) identified in bacterial (A-B) and archaeal (C-D) communities from each habitat in DNA (A-C) and RNA (B-D) libraries.

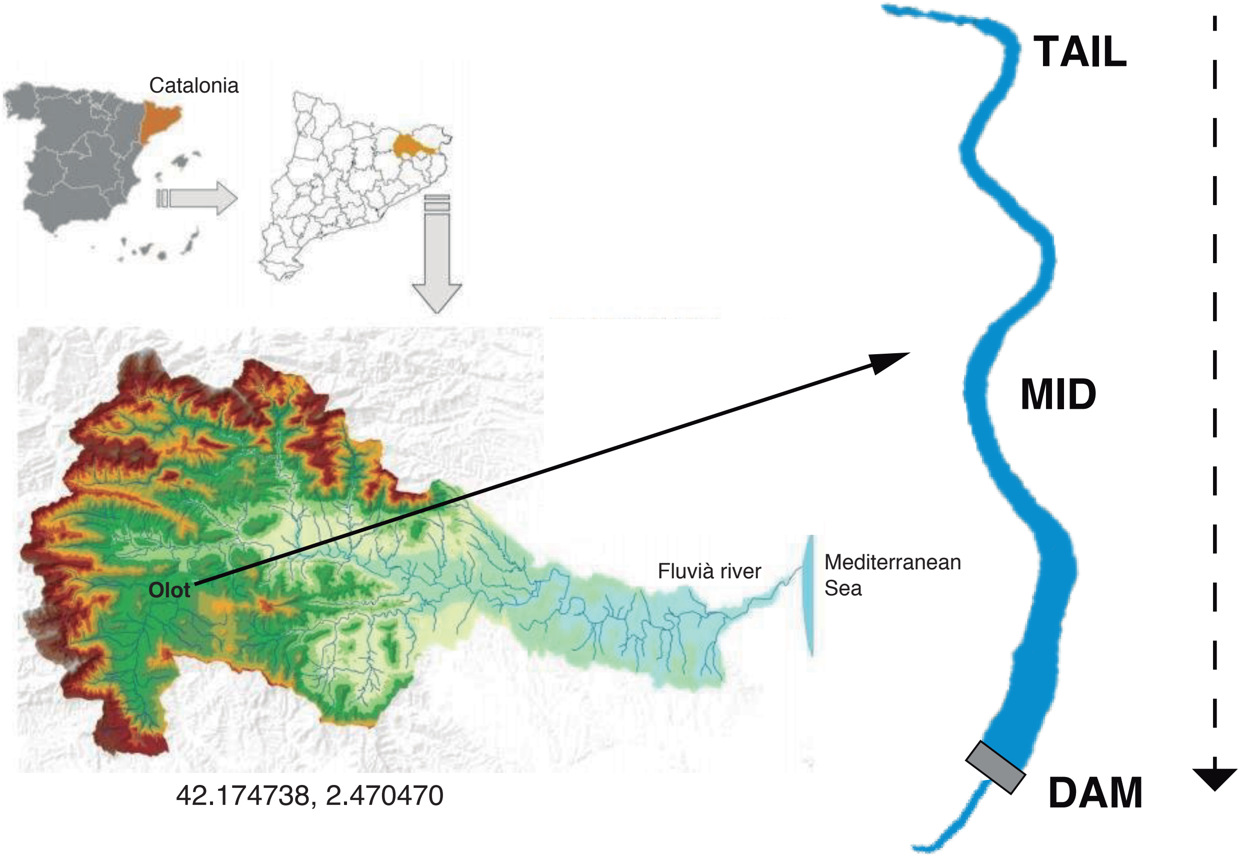


**Supplementary Figure S5.** Top view of the studied stream showing the location of the sampling sites.

**Supplementary Table S1.** Profiles of physical and chemical data in the Olot waterbody at the different sampling sites (tail, mid, dam).

## Tail

| Depth (m) | Temp (ºC) | Conduct. ($\mu$S/cm) | Oxygen (mg/L) | Oxygen (% sat) | pH | PAR ($\mu$E/cm2/s) | Turbidity (NTU) | Chl *a* ($\mu$g/L) |
|---|---|---|---|---|---|---|---|---|
| 0.02 | 17.07 | 523.75 | 9.43 | 97.89 | 8.20 | 209.81 | 6.15 | 2.23 |
| 0.04 | 17.05 | 523.78 | 9.44 | 97.97 | 8.20 | 174.96 | 5.98 | 2.20 |
| 0.06 | 17.04 | 523.78 | 9.46 | 98.13 | 8.20 | 140.66 | 5.91 | 2.18 |
| 0.10 | 17.04 | 524.02 | 9.49 | 98.48 | 8.20 | 170.92 | 6.06 | 2.23 |
| 0.13 | 17.05 | 523.62 | 9.52 | 98.82 | 8.20 | 123.78 | 6.08 | 2.27 |
| 0.16 | 17.04 | 523.44 | 9.54 | 98.98 | 8.20 | 140.07 | 6.28 | 2.27 |
| 0.22 | 17.02 | 523.32 | 9.55 | 99.00 | 8.20 | 85.52 | 6.08 | 2.21 |
| 0.25 | 17.00 | 523.20 | 9.54 | 98.85 | 8.20 | 79.28 | 6.01 | 2.20 |
| 0.27 | 16.98 | 523.13 | 9.51 | 98.53 | 8.20 | 49.25 | 5.83 | 2.23 |
| 0.30 | 16.97 | 523.29 | 9.49 | 98.31 | 8.21 | 41.09 | 5.87 | 2.42 |
| 0.33 | 16.97 | 523.32 | 9.47 | 98.09 | 8.20 | 38.67 | 5.86 | 2.42 |
| 0.35 | 16.97 | 523.20 | 9.45 | 97.90 | 8.21 | 39.31 | 5.95 | 2.38 |
| 0.37 | 16.97 | 523.26 | 9.42 | 97.57 | 8.21 | 36.47 | 6.04 | 2.30 |
| 0.40 | 16.97 | 522.92 | 9.42 | 97.56 | 8.21 | 35.58 | 5.80 | 2.40 |
| 0.42 | 16.96 | 522.92 | 9.41 | 97.50 | 8.21 | 35.03 | 5.92 | 2.46 |
| 0.43 | 16.94 | 522.16 | 9.40 | 97.35 | 8.21 | 34.35 | 6.14 | 2.37 |
| 0.43 | 16.91 | 522.92 | 9.40 | 97.27 | 8.21 | 33.79 | 6.35 | 2.26 |
| 0.45 | 16.91 | 522.77 | 9.38 | 97.03 | 8.21 | 34.56 | 6.29 | 2.25 |
| 0.47 | 16.91 | 521.97 | 9.37 | 96.95 | 8.21 | 33.46 | 6.00 | 2.20 |
| 0.48 | 16.89 | 521.73 | 9.35 | 96.74 | 8.21 | 34.38 | 5.94 | 2.28 |
| 0.49 | 16.86 | 521.67 | 9.36 | 96.74 | 8.21 | 34.57 | 6.24 | 2.22 |
| 0.49 | 16.85 | 521.73 | 9.35 | 96.58 | 8.21 | 34.93 | 6.09 | 2.20 |
| 0.51 | 16.84 | 521.85 | 9.34 | 96.46 | 8.21 | 33.79 | 6.24 | 2.23 |
| 0.54 | 16.84 | 520.45 | 9.32 | 96.29 | 8.21 | 33.93 | 6.44 | 2.25 |
| 0.55 | 16.82 | 519.99 | 9.32 | 96.22 | 8.21 | 33.86 | 6.37 | 2.22 |
| 0.57 | 16.76 | 520.11 | 9.30 | 95.95 | 8.21 | 33.32 | 6.35 | 2.21 |
| 0.58 | 16.73 | 520.97 | 9.28 | 95.69 | 8.21 | 33.48 | 6.50 | 2.31 |
| 0.58 | 16.74 | 521.36 | 9.26 | 95.49 | 8.21 | 33.35 | 6.47 | 2.41 |
| 0.59 | 16.77 | 521.73 | 9.23 | 95.21 | 8.21 | 32.57 | 6.66 | 2.78 |
| 0.60 | 16.80 | 521.61 | 9.20 | 95.01 | 8.21 | 26.24 | 6.58 | 2.66 |
| 0.63 | 16.82 | 521.58 | 9.19 | 94.88 | 8.21 | 20.92 | 6.59 | 2.56 |
| 0.63 | 16.82 | 520.75 | 9.17 | 94.74 | 8.21 | 26.58 | 7.67 | 2.50 |
| 0.63 | 16.80 | 520.32 | 9.18 | 94.72 | 8.21 | 27.23 | 8.61 | 2.38 |
| 0.64 | 16.76 | 520.32 | 9.19 | 94.78 | 8.21 | 26.78 | 6.84 | 2.37 |
| 0.64 | 16.73 | 520.84 | 9.21 | 94.98 | 8.21 | 27.64 | 6.76 | 2.33 |

**Middle**

| Depth (m) | Temp (°C) | Conduct. ($\mu$S/cm) | Oxygen (mg/L) | Oxygen (% sat) | pH | PAR ($\mu$E/cm2/s) | Turbidity (NTU) | Chl *a* ($\mu$g/L) |
|---|---|---|---|---|---|---|---|---|
| 0.057 | 16.80 | 502.25 | 8.63 | 89.12 | 8.209 | 1417.100 | 10.294 | 1.938 |
| 0.094 | 16.71 | 501.70 | 8.49 | 87.49 | 8.209 | 702.290 | 9.722 | 2.037 |
| 0.126 | 16.65 | 501.03 | 8.36 | 86.02 | 8.209 | 609.560 | 9.688 | 2.098 |
| 0.192 | 16.61 | 501.37 | 8.23 | 84.63 | 8.208 | 553.230 | 11.427 | 2.243 |
| 0.261 | 16.59 | 500.08 | 8.12 | 83.39 | 8.208 | 216.100 | 12.476 | 2.136 |
| 0.319 | 16.55 | 497.86 | 8.03 | 82.42 | 8.208 | 203.320 | 13.470 | 2.213 |
| 0.366 | 16.49 | 496.30 | 7.95 | 81.53 | 8.206 | 169.960 | 14.576 | 2.235 |
| 0.383 | 16.42 | 496.88 | 7.88 | 80.71 | 8.207 | 137.320 | 13.811 | 2.235 |
| 0.405 | 16.38 | 498.68 | 7.83 | 80.08 | 8.209 | 170.950 | 13.127 | 2.327 |
| 0.465 | 16.37 | 499.20 | 7.79 | 79.67 | 8.206 | 202.500 | 12.362 | 2.335 |
| 0.511 | 16.36 | 501.37 | 7.75 | 79.23 | 8.207 | 196.610 | 12.221 | 2.266 |
| 0.541 | 16.32 | 501.82 | 7.71 | 78.76 | 8.206 | 232.240 | 11.069 | 2.297 |
| 0.569 | 16.28 | 501.85 | 7.69 | 78.51 | 8.205 | 253.070 | 10.925 | 2.327 |
| 0.597 | 16.25 | 501.73 | 7.65 | 78.06 | 8.206 | 296.990 | 10.872 | 2.251 |
| 0.622 | 16.24 | 501.24 | 7.63 | 77.82 | 8.209 | 297.250 | 11.975 | 2.281 |
| 0.639 | 16.22 | 501.06 | 7.61 | 77.63 | 8.208 | 368.950 | 12.406 | 2.228 |
| 0.657 | 16.22 | 501.12 | 7.59 | 77.37 | 8.209 | 551.580 | 12.606 | 2.251 |
| 0.711 | 16.23 | 501.24 | 7.58 | 77.35 | 8.207 | 422.320 | 12.905 | 2.213 |
| 0.778 | 16.22 | 501.03 | 7.58 | 77.34 | 8.205 | 523.720 | 12.337 | 2.235 |
| 0.831 | 16.20 | 500.60 | 7.59 | 77.33 | 8.206 | 393.520 | 12.430 | 2.297 |
| 0.896 | 16.18 | 500.48 | 7.60 | 77.40 | 8.205 | 377.940 | 13.287 | 2.258 |
| 0.973 | 16.17 | 500.36 | 7.61 | 77.55 | 8.204 | 354.530 | 14.187 | 2.266 |
| 1.058 | 16.16 | 501.27 | 7.61 | 77.51 | 8.204 | 274.750 | 14.099 | 2.358 |
| 1.160 | 16.15 | 502.83 | 7.61 | 77.49 | 8.204 | 226.480 | 14.275 | 2.373 |
| 1.267 | 16.13 | 503.59 | 7.61 | 77.47 | 8.200 | 173.040 | 13.842 | 2.472 |
| 1.367 | 16.12 | 503.56 | 7.60 | 77.30 | 8.203 | 137.730 | 14.427 | 2.380 |
| 1.447 | 16.10 | 504.51 | 7.58 | 77.13 | 8.203 | 115.870 | 14.145 | 2.556 |
| 1.505 | 16.09 | 504.82 | 7.56 | 76.91 | 8.202 | 102.170 | 14.290 | 2.480 |
| 1.567 | 16.07 | 505.00 | 7.55 | 76.71 | 8.203 | 90.460 | 14.761 | 2.411 |
| 1.634 | 16.06 | 505.18 | 7.53 | 76.53 | 8.200 | 71.624 | 15.045 | 2.403 |
| 1.670 | 16.05 | 505.33 | 7.53 | 76.53 | 8.202 | 68.848 | 15.011 | 2.335 |
| 1.682 | 16.04 | 505.27 | 7.55 | 76.69 | 8.201 | 67.128 | 14.883 | 2.327 |
| 1.710 | 16.04 | 505.33 | 7.57 | 76.92 | 8.201 | 65.119 | 14.794 | 2.304 |
| 1.744 | 16.04 | 505.61 | 7.60 | 77.15 | 8.204 | 61.852 | 15.324 | 2.304 |
| 1.781 | 16.03 | 505.70 | 7.62 | 77.41 | 8.203 | 57.655 | 15.286 | 2.403 |
| 1.832 | 16.03 | 505.98 | 7.65 | 77.69 | 8.202 | 52.881 | 16.297 | 2.396 |
| 1.870 | 16.02 | 506.16 | 7.67 | 77.84 | 8.204 | 49.163 | 16.091 | 2.480 |
| 1.898 | 16.02 | 506.04 | 7.69 | 78.03 | 8.202 | 47.091 | 16.056 | 2.457 |
| 1.937 | 16.01 | 506.28 | 7.69 | 78.04 | 8.204 | 43.757 | 15.352 | 2.571 |
| 2.000 | 16.01 | 506.46 | 7.69 | 78.04 | 8.206 | 39.145 | 15.644 | 2.510 |
| 2.060 | 16.00 | 506.37 | 7.69 | 78.03 | 8.204 | 35.260 | 15.867 | 2.464 |
| 2.095 | 15.99 | 506.40 | 7.69 | 78.02 | 8.206 | 33.538 | 15.295 | 2.472 |
| 2.117 | 15.99 | 506.40 | 7.68 | 77.92 | 8.207 | 32.312 | 15.549 | 2.403 |

| | | | | | | | | |
|---|---|---|---|---|---|---|---|---|
| 2.149 | 15.99 | 506.37 | 7.68 | 77.96 | 8.203 | 30.610 | 15.223 | 2.388 |
| 2.202 | 15.99 | 506.52 | 7.67 | 77.83 | 8.203 | 29.295 | 15.551 | 2.495 |
| 2.250 | 15.99 | 506.59 | 7.68 | 77.95 | 8.207 | 26.775 | 15.486 | 2.411 |
| 2.255 | 15.98 | 506.52 | 7.69 | 78.04 | 8.205 | 26.490 | 15.747 | 2.411 |
| 2.270 | 15.98 | 506.52 | 7.70 | 78.09 | 8.207 | 25.864 | 15.318 | 2.388 |
| 2.322 | 15.98 | 506.52 | 7.67 | 77.79 | 8.204 | 23.478 | 16.051 | 2.441 |
| 2.395 | 15.98 | 506.59 | 7.68 | 77.93 | 8.208 | 21.074 | 15.612 | 2.548 |
| 2.470 | 15.98 | 506.62 | 7.65 | 77.62 | 8.206 | 18.806 | 15.457 | 2.686 |
| 2.516 | 15.98 | 506.59 | 7.66 | 77.72 | 8.208 | 17.813 | 15.108 | 2.701 |
| 2.542 | 15.97 | 506.62 | 7.69 | 77.98 | 8.207 | 17.219 | 14.853 | 2.655 |
| 2.569 | 15.97 | 506.62 | 7.70 | 78.08 | 8.207 | 16.827 | 14.683 | 2.556 |
| 2.600 | 15.97 | 506.65 | 7.71 | 78.18 | 8.207 | 16.093 | 14.694 | 2.525 |
| 2.627 | 15.96 | 506.68 | 7.72 | 78.33 | 8.211 | 15.344 | 14.527 | 2.441 |
| 2.651 | 15.96 | 506.68 | 7.74 | 78.46 | 8.209 | 14.776 | 14.750 | 2.441 |
| 2.674 | 15.96 | 506.71 | 7.74 | 78.53 | 8.209 | 14.228 | 14.872 | 2.548 |
| 2.695 | 15.96 | 506.68 | 7.75 | 78.57 | 8.206 | 13.734 | 15.457 | 2.686 |
| 2.717 | 15.96 | 506.71 | 7.73 | 78.44 | 8.209 | 13.200 | 15.763 | 2.731 |
| 2.736 | 15.96 | 506.62 | 7.72 | 78.25 | 8.208 | 12.757 | 15.682 | 2.609 |
| 2.755 | 15.96 | 506.68 | 7.71 | 78.18 | 8.211 | 12.287 | 15.492 | 2.503 |
| 2.779 | 15.96 | 506.71 | 7.71 | 78.17 | 8.211 | 11.815 | 14.992 | 2.457 |
| 2.807 | 15.96 | 506.74 | 7.70 | 78.11 | 8.209 | 11.307 | 15.246 | 2.441 |
| 2.829 | 15.96 | 506.74 | 7.69 | 78.02 | 8.211 | 11.073 | 15.686 | 2.525 |
| 2.850 | 15.96 | 506.74 | 7.70 | 78.05 | 8.211 | 10.890 | 16.070 | 2.579 |
| 2.875 | 15.96 | 506.68 | 7.69 | 78.03 | 8.212 | 10.362 | 16.575 | 2.617 |
| 2.887 | 15.96 | 506.74 | 7.69 | 77.95 | 8.211 | 10.253 | 16.205 | 2.663 |
| 2.903 | 15.96 | 506.74 | 7.67 | 77.82 | 8.213 | 10.053 | 15.768 | 2.571 |
| 2.931 | 15.96 | 506.74 | 7.67 | 77.81 | 8.212 | 9.564 | 15.740 | 2.380 |
| 2.954 | 15.96 | 506.71 | 7.67 | 77.77 | 8.212 | 9.328 | 16.209 | 2.510 |
| 2.997 | 15.96 | 506.74 | 7.66 | 77.72 | 8.211 | 8.721 | 16.363 | 2.419 |
| 3.066 | 15.96 | 506.74 | 7.66 | 77.71 | 8.209 | 7.881 | 17.506 | 2.396 |
| 3.137 | 15.96 | 506.80 | 7.67 | 77.82 | 8.212 | 7.207 | 18.425 | 2.518 |
| 3.187 | 15.96 | 506.80 | 7.68 | 77.91 | 8.209 | 6.779 | 18.704 | 2.655 |
| 3.232 | 15.96 | 506.86 | 7.69 | 77.96 | 8.211 | 6.402 | 19.440 | 3.365 |
| 3.281 | 15.96 | 506.92 | 7.69 | 77.96 | 8.213 | 6.159 | 19.537 | 3.243 |
| 3.343 | 15.96 | 506.92 | 7.68 | 77.90 | 8.213 | 5.657 | 18.727 | 2.998 |
| 3.400 | 15.96 | 506.89 | 7.68 | 77.87 | 8.211 | 5.246 | 18.458 | 2.823 |
| 3.451 | 15.96 | 506.98 | 7.69 | 78.00 | 8.213 | 4.882 | 19.850 | 2.815 |
| 3.499 | 15.96 | 506.95 | 7.67 | 77.78 | 8.213 | 4.536 | 20.233 | 2.792 |
| 3.552 | 15.96 | 506.98 | 7.66 | 77.72 | 8.213 | 4.208 | 20.739 | 2.564 |
| 3.606 | 15.96 | 506.98 | 7.65 | 77.62 | 8.210 | 3.878 | 21.118 | 2.625 |
| 3.640 | 15.96 | 506.95 | 7.64 | 77.50 | 8.213 | 3.737 | 21.008 | 2.647 |
| 3.662 | 15.96 | 506.92 | 7.63 | 77.34 | 8.211 | 3.602 | 22.587 | 2.548 |
| 3.723 | 15.96 | 507.01 | 7.60 | 77.08 | 8.211 | 3.217 | 25.645 | 2.640 |
| 3.792 | 15.96 | 507.17 | 7.59 | 76.93 | 8.211 | 2.892 | 28.449 | 2.655 |
| 3.821 | 15.96 | 507.29 | 7.56 | 76.71 | 8.211 | 2.859 | 26.707 | 2.640 |
| 3.826 | 15.96 | 507.23 | 7.56 | 76.64 | 8.209 | 2.834 | 26.682 | 2.556 |

| | | | | | | | | |
|---|---|---|---|---|---|---|---|---|
| 3.833 | 15.96 | 507.10 | 7.55 | 76.52 | 8.210 | 2.799 | 27.626 | 2.564 |
| 3.843 | 15.96 | 507.17 | 7.53 | 76.40 | 8.210 | 2.745 | 27.617 | 2.579 |
| 3.852 | 15.96 | 507.23 | 7.53 | 76.38 | 8.210 | 2.672 | 27.777 | 2.525 |
| 3.861 | 15.96 | 507.23 | 7.52 | 76.29 | 8.212 | 2.584 | 28.756 | 2.541 |
| 3.867 | 15.96 | 507.23 | 7.50 | 76.09 | 8.210 | 2.528 | 28.252 | 2.770 |
| 3.873 | 15.96 | 507.23 | 7.49 | 75.96 | 8.209 | 2.476 | 26.606 | 2.945 |
| 3.875 | 15.96 | 507.23 | 7.46 | 75.66 | 8.210 | 2.468 | 27.571 | 2.853 |
| 3.876 | 15.96 | 507.17 | 7.43 | 75.37 | 8.209 | 2.444 | 28.824 | 2.815 |

## Dam

| Depth (m) | Temp (ºC) | Conduct. (μS/cm) | Oxygen (mg/L) | Oxygen (% sat) | pH | PAR (μE/cm2/s) | Turbidity (NTU) | Chl *a* (μg/L) |
|---|---|---|---|---|---|---|---|---|
| 0.028 | 16.978 | 453.084 | 6.7467 | 69.8828 | 8.12 | 68.162 | 13.573 | 2.358 |
| 0.06 | 16.9385 | 452.78 | 6.75343 | 69.8949 | 8.12 | 68.39 | 13.403 | 2.426 |
| 0.087 | 16.9039 | 453.297 | 6.7519 | 69.8287 | 8.12 | 68.534 | 13.733 | 2.411 |
| 0.097 | 16.8978 | 452.841 | 6.7403 | 69.6998 | 8.13 | 68.246 | 14.137 | 2.51 |
| 0.101 | 16.889 | 452.902 | 6.73527 | 69.635 | 8.13 | 67.911 | 14.105 | 2.51 |
| 0.107 | 16.8813 | 453.084 | 6.71825 | 69.4479 | 8.13 | 67.697 | 14.809 | 2.556 |
| 0.108 | 16.8844 | 453.176 | 6.7084 | 69.3506 | 8.13 | 67.365 | 14.41 | 2.556 |
| 0.139 | 16.8914 | 453.267 | 6.70129 | 69.2872 | 8.13 | 65.983 | 13.5 | 2.541 |
| 0.147 | 16.8986 | 453.084 | 6.69891 | 69.273 | 8.12 | 67.756 | 13.277 | 2.541 |
| 0.182 | 16.9018 | 453.785 | 6.68977 | 69.1834 | 8.12 | 67.507 | 13.512 | 2.556 |
| 0.218 | 16.9221 | 453.145 | 6.6967 | 69.2841 | 8.12 | 63.314 | 15.084 | 2.51 |
| 0.229 | 16.9192 | 451.227 | 6.70862 | 69.4028 | 8.12 | 64.199 | 14.591 | 2.586 |
| 0.246 | 16.8548 | 452.567 | 6.73258 | 69.5575 | 8.12 | 63.593 | 14.717 | 2.51 |
| 0.264 | 16.8363 | 452.78 | 6.74607 | 69.6699 | 8.13 | 63.873 | 14.191 | 2.525 |
| 0.288 | 16.8485 | 451.958 | 6.74084 | 69.6335 | 8.13 | 24.382 | 14.605 | 2.571 |
| 0.332 | 16.8314 | 451.745 | 6.73774 | 69.5765 | 8.12 | 26.037 | 14.578 | 2.48 |
| 0.367 | 16.8024 | 451.106 | 6.72369 | 69.3893 | 8.12 | 22.469 | 13.987 | 2.632 |
| 0.39 | 16.7681 | 451.014 | 6.71089 | 69.2075 | 8.12 | 21.432 | 14.168 | 2.609 |
| 0.415 | 16.7376 | 450.984 | 6.70167 | 69.0685 | 8.13 | 18.892 | 14.212 | 2.602 |
| 0.463 | 16.7193 | 450.984 | 6.68468 | 68.867 | 8.13 | 16.718 | 13.954 | 2.594 |
| 0.489 | 16.7074 | 449.949 | 6.6816 | 68.8179 | 8.12 | 16.318 | 14.237 | 2.632 |
| 0.533 | 16.6726 | 450.284 | 6.69132 | 68.8679 | 8.12 | 15.006 | 14.408 | 2.571 |
| 0.612 | 16.6474 | 449.949 | 6.7099 | 69.0225 | 8.12 | 13.581 | 14.473 | 2.625 |
| 0.652 | 16.6196 | 448.153 | 6.7163 | 69.0478 | 8.12 | 13.265 | 14.351 | 2.602 |
| 0.674 | 16.5528 | 448.336 | 6.73286 | 69.1213 | 8.12 | 12.677 | 14.229 | 2.571 |
| 0.719 | 16.4879 | 448.64 | 6.72806 | 68.978 | 8.13 | 11.978 | 14.208 | 2.67 |
| 0.777 | 16.45 | 449.828 | 6.7181 | 68.8214 | 8.12 | 11.171 | 15.65 | 2.533 |
| 0.825 | 16.4412 | 449.554 | 6.69416 | 68.5634 | 8.12 | 10.774 | 16.022 | 2.594 |
| 0.877 | 16.4364 | 449.432 | 6.68304 | 68.4426 | 8.13 | 10.02 | 15.452 | 2.609 |
| 0.924 | 16.4212 | 451.289 | 6.67162 | 68.3043 | 8.12 | 9.596 | 15.827 | 2.64 |
| 0.977 | 16.4198 | 451.441 | 6.67524 | 68.3392 | 8.12 | 8.9684 | 15.553 | 2.663 |
| 1.023 | 16.4321 | 451.441 | 6.6756 | 68.3606 | 8.12 | 8.5365 | 15.259 | 2.731 |
| 1.073 | 16.4409 | 451.624 | 6.68709 | 68.491 | 8.12 | 8.2316 | 16.104 | 2.678 |

| | | | | | | | | |
|---|---|---|---|---|---|---|---|---|
| 1.128 | 16.441 | 452.567 | 6.69855 | 68.6088 | 8.12 | 7.6273 | 15.637 | 2.663 |
| 1.19 | 16.4278 | 455.369 | 6.72458 | 68.8569 | 8.12 | 7.0402 | 15.579 | 2.647 |
| 1.264 | 16.3955 | 454.942 | 6.74043 | 68.9724 | 8.12 | 6.297 | 15.673 | 2.655 |
| 1.332 | 16.3665 | 455.003 | 6.75544 | 69.0837 | 8.12 | 5.7584 | 17.456 | 2.647 |
| 1.4 | 16.346 | 457.013 | 6.76453 | 69.1474 | 8.12 | 5.196 | 18.177 | 2.67 |
| 1.469 | 16.3328 | 457.378 | 6.78075 | 69.2941 | 8.12 | 4.6926 | 19.169 | 2.67 |
| 1.527 | 16.325 | 459.236 | 6.78535 | 69.3301 | 8.12 | 4.355 | 19.936 | 2.67 |
| 1.589 | 16.3209 | 462.83 | 6.7974 | 69.4482 | 8.12 | 4.0113 | 22.429 | 2.693 |
| 1.656 | 16.3202 | 469.105 | 6.79539 | 69.4283 | 8.12 | 3.6342 | 21.391 | 2.647 |
| 1.717 | 16.3211 | 472.823 | 6.78663 | 69.341 | 8.12 | 3.3521 | 21.361 | 2.586 |
| 1.782 | 16.32 | 473.432 | 6.77173 | 69.1872 | 8.12 | 3.203 | 19.533 | 2.571 |
| 1.851 | 16.3172 | 475.109 | 6.73681 | 68.8268 | 8.11 | 2.8649 | 20.031 | 2.724 |
| 1.897 | 16.3138 | 474.743 | 6.69953 | 68.4409 | 8.11 | 2.7106 | 19.707 | 2.655 |
| 1.942 | 16.3099 | 474.987 | 6.66356 | 68.068 | 8.12 | 2.5289 | 19.684 | 2.701 |
| 1.998 | 16.3063 | 475.048 | 6.66258 | 68.0528 | 8.11 | 2.2727 | 19.337 | 2.609 |
| 2.056 | 16.3033 | 474.53 | 6.68737 | 68.3017 | 8.12 | 2.04 | 19.442 | 2.564 |
| 2.088 | 16.2993 | 474.926 | 6.74337 | 68.8679 | 8.12 | 1.9813 | 18.397 | 2.51 |
| 2.106 | 16.2958 | 475.109 | 6.82744 | 69.7214 | 8.12 | 1.8922 | 18.063 | 2.548 |
| 2.133 | 16.2938 | 475.566 | 6.91189 | 70.5809 | 8.12 | 1.8218 | 18.061 | 2.503 |
| 2.161 | 16.2917 | 475.749 | 6.99959 | 71.4732 | 8.12 | 1.6925 | 18.017 | 2.518 |
| 2.206 | 16.2903 | 475.657 | 7.07534 | 72.2446 | 8.12 | 1.5774 | 18.221 | 2.503 |
| 2.245 | 16.29 | 475.688 | 7.13068 | 72.8092 | 8.12 | 1.509 | 18.786 | 2.472 |
| 2.263 | 16.29 | 475.535 | 7.17961 | 73.3088 | 8.12 | 1.4843 | 19.278 | 2.51 |
| 2.277 | 16.2899 | 475.352 | 7.19592 | 73.4751 | 8.12 | 1.4467 | 18.475 | 2.487 |
| 2.325 | 16.2903 | 475.352 | 7.2105 | 73.6246 | 8.12 | 1.3396 | 18.433 | 2.51 |
| 2.389 | 16.2912 | 477.09 | 7.20519 | 73.5723 | 8.12 | 1.1957 | 18.172 | 2.541 |
| 2.439 | 16.2875 | 477.852 | 7.20729 | 73.5882 | 8.13 | 1.1034 | 17.92 | 2.831 |
| 2.482 | 16.2805 | 477.852 | 7.20416 | 73.5454 | 8.12 | 1.0435 | 17.746 | 2.701 |
| 2.534 | 16.2752 | 476.846 | 7.18919 | 73.384 | 8.12 | 0.95154 | 17.496 | 2.762 |
| 2.587 | 16.272 | 475.413 | 7.18488 | 73.3348 | 8.12 | 0.88215 | 17.35 | 2.792 |
| 2.635 | 16.2711 | 475.231 | 7.1703 | 73.1846 | 8.13 | 0.82693 | 17.489 | 2.754 |
| 2.673 | 16.2714 | 475.353 | 7.17035 | 73.1855 | 8.13 | 0.79056 | 17.327 | 2.747 |
| 2.705 | 16.2722 | 475.566 | 7.16389 | 73.1209 | 8.13 | 0.77584 | 17.348 | 2.701 |
| 2.721 | 16.273 | 475.231 | 7.15647 | 73.0463 | 8.13 | 0.7509 | 18.069 | 2.594 |
| 2.723 | 16.2734 | 475.109 | 7.12526 | 72.7283 | 8.13 | 0.76233 | 18.397 | 2.579 |
| 2.726 | 16.2735 | 475.17 | 7.07213 | 72.1863 | 8.13 | 0.74971 | 18.036 | 2.441 |
